# Supplementary material for: Assignment of the somatic A/B compartments to chromatin domains in giant transcriptionally active lampbrush chromosomes
Source: Epigenetics Chromatin. 2023 Jun 15;16:24. doi: 10.1186/s13072-023-00499-2 (PMC10268536; doi:10.1186/s13072-023-00499-2)
Supplement: Supplementary file 1 — Additional file 1: Figure S1. Distribution of A/B compartments along the chicken chromosomes 3, 5 and 6 in embryonic fibroblasts compared with the chromomeric pattern in the corresponding lampbrush chromosomes. Figure S2. Examples of constitutive B compartment regions in previously studied chicken cell types. Table S1. Genomic regions mapped on chicken lampbrush chromosomes and marked on the coordinate line on Figures 1, 2, 3, 4, 5, Additional file 1: Figure S1, according to the chicken genome version 5 (galGal5). Table S2. The list of BAC clones containing fragments of chicken genomic DNA from the CHORI-261 library that were used as DNA-probes for FISH; coordinates are indicated according to the chicken genome version 5 (galGal5). [file 13072_2023_499_MOESM1_ESM.pdf]

Additional Materials

Assignment of the somatic A/B compartments to chromatin domains in giant transcriptionally active lampbrush chromosomes

Alla Krasikova[1] *, Tatiana Kulikova[1] , Juan Sebastian Rodriguez Ramos[1] , Antonina Maslova[1]

[1] Saint-Petersburg State University, Saint-Petersburg, Russia
* a.krasikova@spbu.ru

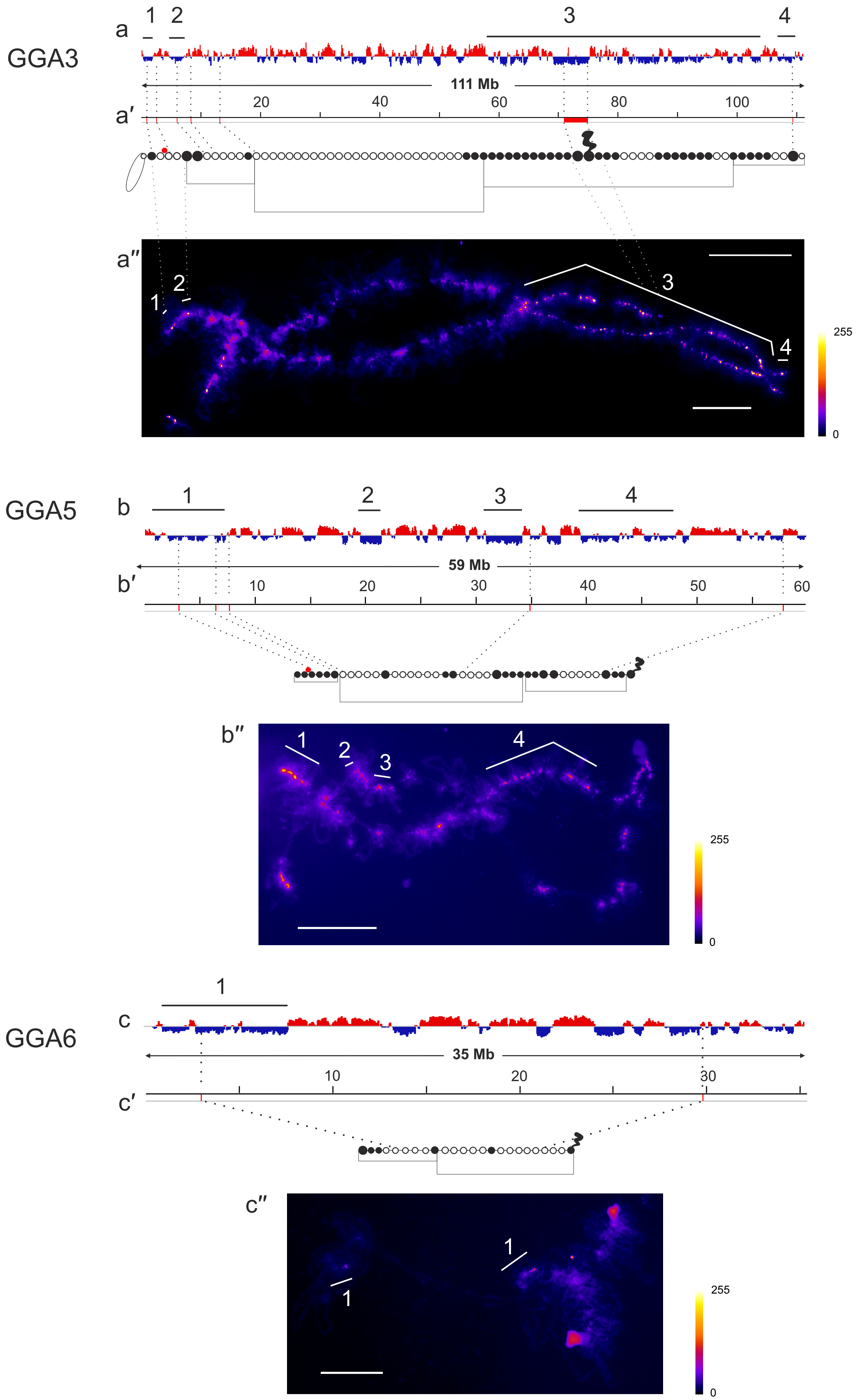

GGA3
a
1
2
3
4
111 Mb
20
40
60
80
100
a′
a″
255
0
GGA5
b
59 Mb
10
20
30
40
50
60
b′
b″
GGA6
c
35 Mb
10
20
30
c′
c″

Figure S1. Distribution of A/B compartments along the chicken chromosomes 3, 5 and 6 in embryonic fibroblasts compared with the chromomeric pattern in the corresponding lampbrush chromosomes.

Distribution of A (red) and B (dark blue) compartments along the chicken chromosomes 3 (GGA3) (**a**), 5 (GGA5) (**b**) and 6 (GGA6) (**c**) in embryonic fibroblasts viewed by Integrative Genomics Viewer (IGV) (according to [15]); cytological maps of chicken lampbrush chromosomes 3 (**a′**), 5 (**b′**), 6 (**c′**) depicting DAPI-staining pattern of chromomeres and relative contour length of lateral loops, black circles – dense chromomeres brightly stained with DAPI (according to [53, 54]). Dotted lines connect the genomic positions of the BAC-clones (Additional Table 1) with their positions on the cytological maps. **a″**-**c″** – lampbrush chromosomes 3 (**a″**), 5 (**b″**), 6 (**c″**) stained with DAPI, pixel intensities displayed with multicolored ImageJ look-up table, numbered lines on **a**-**c** and **a″**-**c″** indicate positions of chromomere clusters brightly stained with DAPI. Scale bars: 20 μm.

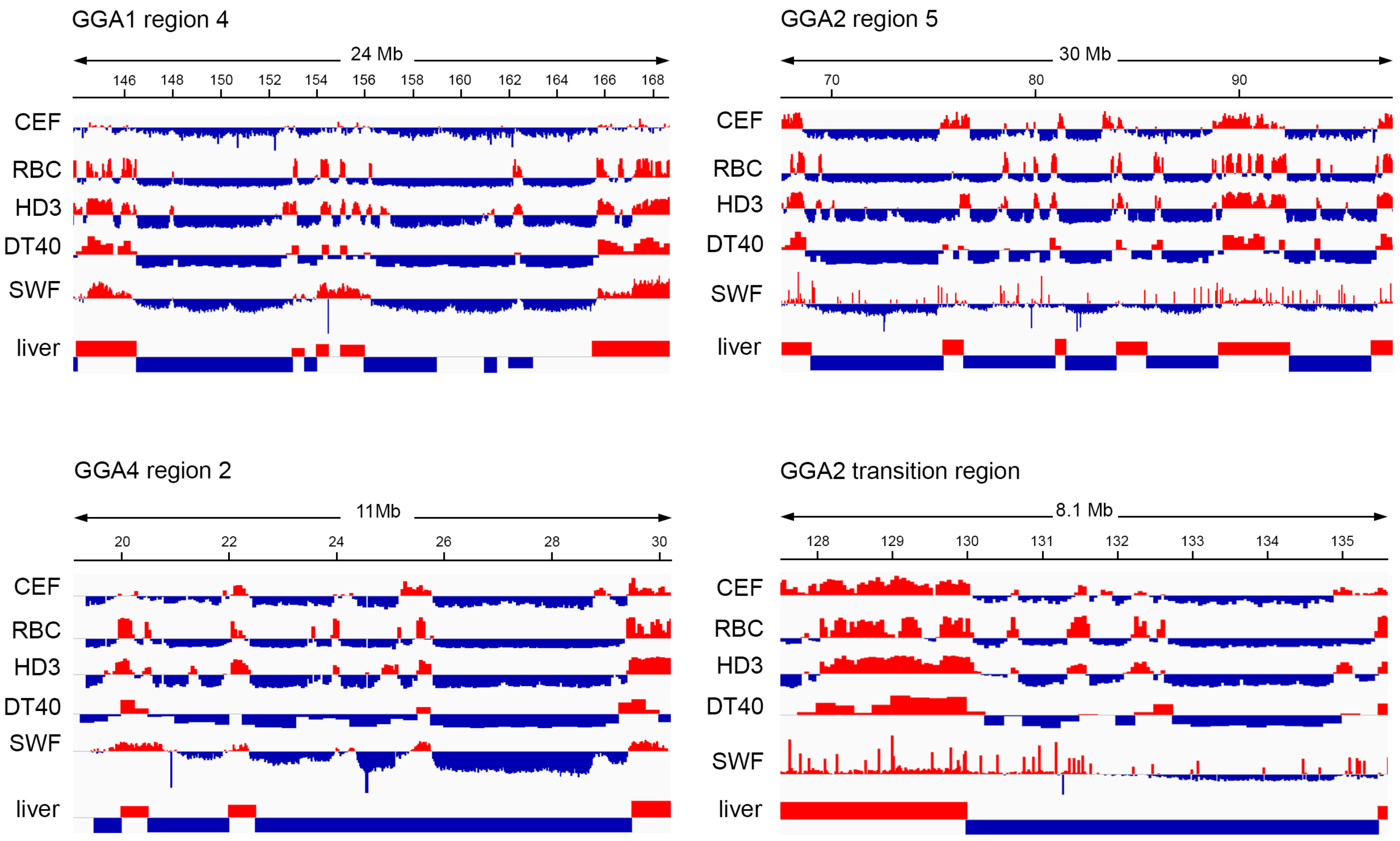
GGA1 region 4
24 Mb
146
148
150
152
154
156
158
160
162
164
166
168
CEF
RBC
HD3
DT40
SWF
liver
GGA2 region 5
30 Mb
70
80
90
CEF
RBC
HD3
DT40
SWF
liver
GGA4 region 2
11Mb
20
22
24
26
28
30
CEF
RBC
HD3
DT40
SWF
liver
GGA2 transition region
8.1 Mb
128
129
130
131
132
133
134
135
CEF
RBC
HD3
DT40
SWF
liver

Figure S2. Examples of constitutive B compartment regions in previously studied chicken cell types.

The regions of large constitutive B compartment domains on chromosomes GGA1, GGA2 and GGA4 and the conservative A-to-B compartment transition region on GGA2. The profiles of A (red) and B (blue) compartment for chicken embryonic fibroblasts (CEF, resolution – 50 Kb), chicken erythrocytes (RBC, resolution – 50 Kb), HD3 erythroblasts (resolution – 50 Kb), DT40 cells (resolution – 250 Kb), small white follicle granulosa cells (SWF, resolution – 20 Kb), and liver cells (resolution – 500 Kb) are plotted in the Integrative Genomics Viewer (IGV).

Table S1. Genomic regions mapped on chicken lampbrush chromosomes and marked on the coordinate line on **Figures 1-5**, **Figure S1**, according to the chicken genome version 5 (galGal5).

Chromosome	DNA-probe	Genetic marker	Genomic coordinates (bp)		Reference
GGA1	W030P07	MCW248	3042685	3042903	[53]
GGA1	W030B21	LEI194	26981390	26981528	[53]
GGA1	CH261-87J17		50659355	50900102	*current study*
GGA1	CH261-54H10		52008845	52206496	*current study*
GGA1	WAG31B10	LEI146	53274224	53274474	[53, 54]
GGA1	WAG13E20	GCT0049	60975037	60975254	[54]
GGA1	WAG69C11	MCW007	63858174	63858471	[54]
GGA1	WAG67J15	LDHB	67084357	67094049	[54]
GGA1	CH261-162E14		70424086	70619427	*current study*
GGA1	CH261-33I15		70675137	70847856	*current study*
GGA1	WAG53E23	LEI0071	76397821	76397841	[54]
GGA1	WAG25G16	LEI101	81210437	81210594	[54]
GGA1	P1H9	GCT13	110607927	110608236	[53]
GGA1	CH261-191J12		147563186	147752805	[39]
GGA1	CH261-54J7		185317106	185519653	*current study*
GGA1	CH261-120O20		185970069	186181883	*current study*
GGA2	W055L19	ADL228	830924	830943	[53]
GGA2	Spaghetti marker		10300000	15000000	[35]
GGA2	CH261-96F4		26339255	26564682	[39]
GGA2	W026B13	MCW63	38085213	38085346	[53]
GGA2	CH261-163C1		39010879	39197698	*current study*
GGA2	CH261-134B20		39844735	40041305	*current study*
GGA2	CH261-182I12		46712094	46921822	[39]
GGA2	W014J06	MCW358	60810746	60810764	[54]
GGA2	P2E4	GCT23	78032760	78032777	[53]
GGA2	W041C02	LEI147	99097193	99096954	[53, 54]
GGA2	CH261-98G7		128115147	128367991	*current study*
GGA2	CH261-54I9		134678478	134876251	*current study*
GGA2	LL2R		142822175	142893497	[65]
GGA3	WAG29L12	MCW0261	835733	835983	[54]
GGA3	WAG54M22	MCW0141	2558467	2558696	[54]
GGA3	WAG13D11	CZ566991	5944007	5944620	[54]
GGA3	WAG40J15	CZ564186	8278622	8279152	[54]
GGA3	WAG32A13	CZ562529	13128312	13128887	[54]
GGA3	chromomere #16-7		71000000	750000000	[35]
GGA3	W013L14	MCW37	109303638	109303891	[53]
GGA4	W008H20	ADL143	1431102	1431253	[53]
GGA4	CH261-33C6		6419141	6610711	[39]
GGA4	W023I16	ADL203	8314125	8314310	[53]
GGA4	W125P16	MCW295	16406325	16406429	[53]
GGA4	CH261-30O11		34089061	34311128	*current study*
GGA4	CH261-49F17		37602285	37797874	[39]
GGA4	CH261-47F11		41547668	41747405	[39]
GGA4	CH261-139O9		62817075	62995554	[39]
GGA4	W012C06	MCW180	72199515	72199534	[53]
GGA4	W013E02	LEI63	82180055	82179854	[53]
GGA4	W037E19	LEI73	85792844	85793001	[53]
GGA5	231H07		3057165	3057166	[56]
GGA5	429G11		6454339	6454340	[56]
GGA5	W037H20	MCW263	7666396	7666785	[53]
GGA5	W003K18	MCW210	34881129	34881612	[53]
GGA5	W009B13	ADL298	57799040	57799394	[53]
GGA6	W027G19	LEI192	2943525	2943846	[53]
GGA6	W010H24	ADL142	29784025	29784326	[53]
GGA7	CH261-93F1		12820752	13020947	*current study*
GGA7	CH261-126G14		13125892	13385143	*current study*
GGA7	CH261-38J23		13436411	13615182	*current study*
GGA14	CH261-94D13		1164728	1385107	*current study*
GGA14	CH261-168C19		1620727	1848725	*current study*
GGA14	WAG32F10		3695102	3695437	[54]
GGA14	CH261-177N7		10232089	10454271	*current study*
GGA14	CH261-99K17		11716239	11911222	*current study*
GGA14	WAG19G22		12851870	12852527	[54]
GGA14	CH261-152F2		13090203	13310067	*current study*
GGA14	WAG42M3		13831988	13832434	[54]

Table S2. The list of BAC clones containing fragments of chicken genomic DNA from the CHORI-261 library that were used as DNA-probes for FISH, coordinates are indicated according to the chicken genome version 5 (galGal5)*.

Chromosome region (Mb)	BAC clone name	Start coordinate (bp)	End coordinate (bp)	Insert lenght (bp)	Figure # with the FISH-mapping data
GGA1_50-52	CH261-87J17	50659355	50900102	240748	Figure 1 d
GGA1_50-52	CH261-51C16	50965726	51148987	183262	
GGA1_50-52	CH261-189I21	51149051	51371768	222718	
GGA1_50-52	CH261-178N20	51428246	51636410	208165	
GGA1_50-52	CH261-104F19	51731158	51950181	219024	
GGA1_50-52	CH261-54H10	52008845	52206496	197652	
GGA1_70-71	CH261-162E14	70424086	70619427	195342	Figure 1 e
GGA1_70-71	CH261-33I15	70675137	70847856	172720	
GGA1_70-71	CH261-180H2	70897644	71097854	200211	
GGA1_185-186	CH261-31K17	185317106	185519653	202548	Figure 1 f
GGA1_185-186	CH261-54J7	185647688	185877592	229905	
GGA1_185-186	CH261-120O20	185970069	186181883	211815	
GGA2_39-40	CH261-163C1	39010879	39197698	186820	Figure 2 d
GGA2_39-40	CH261-63A12	39544475	39746185	201711	
GGA2_39-40	CH261-134B20	39844735	40041305	196571	
GGA2_128-135	CH261-98G7	128115147	128367991	252845	Figure 2 e
GGA2_128-135	CH261-135E13	129835347	130046990	211644	
GGA2_128-135	CH261-12O18	130737070	130936225	199156	
GGA2_128-135	CH261-140E6	132515988	132668627	152640	
GGA2_128-135	CH261-177C13	132943066	133156205	213140	
GGA2_128-135	CH261-97C18	133802274	134043084	240811	
GGA2_128-135	CH261-54I9	134678478	134876251	197774	
GGA4_34-37	CH261-30O11	34089061	34311128	222068	Figure 3 d
GGA4_34-37	CH261-38A10	35326117	35549083	222967	
GGA4_34-37	CH261-124F10	35824748	36061806	237059	
GGA4_34-37	CH261-109C8	37052261	37282110	229850	
GGA7_12-14	CH261-93F1	12820752	13020947	200196	Figure 4 d, d′
GGA7_12-14	CH261-126G14	13125892	13385143	259252	
GGA7_12-14	CH261-38J23	13436411	13615182	178772	
GGA14_1-2	CH261-94D13	1164728	1385107	220380	Figure 5 c
GGA14_1-2	CH261-168C19	1620727	1848725	227999	
GGA14_10-13	CH261-177N7	10232089	10454271	222183	Figure 5 d
GGA14_10-13	CH261-36G10	10599836	10804688	204853	
GGA14_10-13	CH261-179I1	10900679	11080896	180218	
GGA14_10-13	CH261-119E18	11168171	11366145	197975	
GGA14_10-13	CH261-78O7	11501758	11675622	173865	
GGA14_10-13	CH261-99K17	11716239	11911222	194984	
GGA14_10-13	CH261-75C12	12023591	12250932	227342	
GGA14_10-13	CH261-75E9	12322420	12523919	201500	
GGA14_10-13	CH261-57E13	12604054	12796309	192256	
GGA14_10-13	CH261-152F2	13090203	13310067	219865	

* A compartment | B compartment | Mixed compartment in chicken embryonic fibroblasts according to [15].
